# Experiences of double-duty caregivers: a scoping review

**DOI:** 10.1093/geront/gnaf238

**Published:** 2025-10-24

**Authors:** Helma Martens, Annerieke Stoop, Lisette Schipper, Irene Muller-Schoof, Robbert Gobbens

**Affiliations:** Academic Collaborative Center Care for Older Adults, Tranzo, Tilburg School of Social and Behavior Sciences, Tilburg University, Tilburg, The Netherlands; Surplus, Breda, The Netherlands; Academic Collaborative Center Care for Older Adults, Tranzo, Tilburg School of Social and Behavior Sciences, Tilburg University, Tilburg, The Netherlands; Surplus, Breda, The Netherlands; Academic Collaborative Center Care for Older Adults, Tranzo, Tilburg School of Social and Behavior Sciences, Tilburg University, Tilburg, The Netherlands; Academic Collaborative Center Care for Older Adults, Tranzo, Tilburg School of Social and Behavior Sciences, Tilburg University, Tilburg, The Netherlands; Faculty of Health, Sports and Social Work, Inholland University of Applied Sciences, Amsterdam, The Netherlands

**Keywords:** Double-duty caregivers, Informal caregiving, Health care professionals, Lived experiences, Workplace support

## Abstract

**Background and Objectives:**

In an aging society, there is an increasing demand for both formal and informal care. This particularly impacts health care professionals who privately care for an ill relative (double-duty caregivers). This study aims to investigate the state of scientific knowledge on their lived experiences.

**Research Design and Methods:**

Scoping review.

**Results:**

Overall, 2,483 articles were screened, of which 36 articles were eventually included. The studies were conducted in various, mostly Western, countries. Double-duty caregivers lived experiences were found to fall across different themes. Because of their professional knowledge, they themselves and others expect them to be involved in the care for ill relatives. Double-duty caregivers experience mental and emotional consequences. At work, they experience presenteeism and make errors. According to literature, double-duty caregivers need a supportive work environment, like an understanding manager and supporting colleagues.

**Discussion and Implications:**

Several responsibilities of double-duty caregivers overlap with those of informal caregivers in general, while others differ. Given their professional knowledge, double-duty caregivers are likely to more frequently monitor and advise for their ill family members. Support at work should be a shared responsibility between employer and employee. Solutions may also emerge from the network of the ill relative and the double-duty caregiver and the health care organization involved in providing care to the ill relative. Double-duty caregivers, employers, and the care organization involved with the ill relative must be aware of the increasing burden on double-duty caregivers and implement measures to support them.

Worldwide, the population aged 65 years and older is expected to more than double by 2050 (United Nations, 2020). With age, the risk of frailty, illness, long-term conditions, or disability increases, driving higher demands for health care (Broese van Groenou & De Boer, 2016; OECD, 2023; Patterson & Freedman, 2025; van der Ploeg & Gobbens, 2024). To manage these growing demands and costs, Western governments are leading the transition in long-term care. To ensure the sustainability of health care, in this transition older adults are increasingly encouraged to live independently for longer, rather than relocating to nursing homes or relying extensively on professional home care. Instead, in this transition older adults are expected to receive informal care from their immediate network (Detaille et al., 2020; Figueroa et al., 2019; Lindt et al., 2020). As a result, governments place increasing emphasis on informal care, creating a growing demand for informal caregivers for older adults living at home.

In addition, population aging has contributed to a labor shortage in the health care sector, both in Europe (European Commission, 2023) and globally (World Health Organization, 2016). By 2030, the estimated shortage of nurses in the European Union will reach 2.3 million (Michel & Ecarnot, 2020). As a result, fewer health care professionals must manage an increasing workload.

A specific group within the health care sector is facing increasing demands due to the growing emphasis on the role of informal caregivers in supporting their loved ones, combined with workforce shortages in the health care sector. This group consists of health care professionals who, in addition to their professional responsibilities, also provide informal care to a loved one with care and support needs (Detaille et al., 2020). In the international, scientific literature these caregivers are referred to as *double-duty caregivers* (Basley et al., 2024; ­Boumans & Dorant, 2014; DePasquale et al., 2016). Employees in the health care sector are more frequently engaged in informal care compared to those outside the health care sector. In the Netherlands, statistics indicate that one in four health care employees provides informal care to someone within their social network, whereas this proportion is one in six across all sectors (Heitink et al., 2017).

In light of population aging and labor market shortages, double-duty caregivers occupy a unique position in addressing the challenges of both informal caregiving and workforce ­constraints. While research on double-duty caregivers is expanding, a thorough synthesis of this unique position remains limited.

Detaille et al. (2020) conducted a scoping review and a qualitative study on double-duty caregivers. They provided valuable insights into the factors affecting their sustainable employability and their coping strategies. Their work primarily focused on job performance and work-related outcomes. Our scoping review extends beyond this by placing a stronger emphasis on the lived experiences of double-duty caregivers across all life domains. Lived experience refers to the direct, personal experience in the everyday world—including emotional impacts, identity challenges, relationship dynamics, and the unique knowledge transfer between professional and personal caregiving roles—shaped through interactions with others and daily activities (Rich et al., 2013). To adequately prepare double-duty caregivers, society, and health care organizations for the increasing pressures double-duty caregivers will face in the coming decades, and to develop appropriate policies and support systems for them, a deeper understanding of their lived experiences and how they navigate their dual responsibilities in this evolving context is essential. Therefore, we conducted a comprehensive investigation into these lived experiences of double-duty caregivers.

## Aim and research question

We aimed to investigate the state of scientific knowledge on double-duty caregivers with a focus on their lived experiences. This resulted in the following research question: What is known from the scientific literature about the lived experiences of double-duty caregivers, who simultaneously play the roles of health care professional as well as provider of informal care to ill relatives, whether as a partner, family member, friend, or acquaintance?

## Method

### Design

To study double-duty caregivers’ lived experiences, a scoping review was conducted. According to Munn et al. (2018), this type of review is useful in clarifying key concepts and identifying the available evidence, related factors, and knowledge gaps. The Prisma checklist for scoping review (PRISMA-ScR) was used to ensure transparent and complete reporting (Tricco et al., 2018). A full list of search strategies can be found in the review preregistration link (https://doi.org/10.17605/OSF.IO/7NZT3).

The research strategy was developed by four authors (H.M., L.S., A.S., R.G.) with the support of an information specialist. The systematic literature search took place in June 2023 and September 2024 using the electronic databases Medline, CINAHL, and PsychINFO in EBSCO. The databases were searched for articles published in English or Dutch from the year 2000 through September 2024 using the following search string:

(TI(nurse* OR ((health*) N2 (professional* OR personnel*)) OR nursing-assistant* OR health-profession*) OR AB(nurse* OR ((healthcare* OR health-care*) N1 (professional* OR personnel*)) OR nursing-assistant* OR ((health*) N2 (professional*)) OR health-profession*)) AND (TI(((double* OR triple*) N1 (dut*)) OR ((informal OR dual OR multiple) N3 (role*)) OR ((combin*) N3 (care*)) OR nurse-famil* OR ((nurse*) N3 (caregiv*) N3 (relative* OR family)) OR ((informal) N3 (care*) N3 (work* OR professional* OR employment*)) OR ((unpaid*) N2 (care OR family) N2 (work))) OR AB(((double* OR triple*) N1 (dut*)) OR ((informal OR dual OR multiple) N3 (role*)) OR ((combin*) N3 (care*)) OR nurse-famil* OR ((nurse*) N3 (caregiv*) N3 (relative* OR family)) OR ((informal) N3 (care*) N3 (work* OR professional* OR employment*)) OR ((unpaid*) N2 (care OR family) N2 (work))))

### Selection

Articles were eligible for inclusion if they met the following inclusion criteria:

- The study focused on people who work as health care professionals and take care of ill relatives. An *ill relative* refers to a frail relative or a relative with an illness, condition, or disability. Also studies about triple-duty caregivers, that is, double-duty caregivers who care for both their children and older adults (DePasquale et al., 2018d; Häusler et al., 2017) are included in this study.
- The study focused on the (positive or negative) experiences with the double tasks of double-duty caregivers.
- The study included a clear definition of *informal care*, *family care*, or *double-duty caregiving* so that it was clear that informal caregiving involves taking care of a relative with a disease, disorder, or disability.
- The study concerned original, empirical research published in a peer-reviewed scientific journal.
- The study was written in English or Dutch.

Studies focusing on the provision of care for, e.g., healthy children or one’s household (in combination with working as a formal caregiver) or solely on the theoretical aspects of double-duty caregivers, non-scientific papers (e.g., editorials), and studies not focusing on subjective experiences, such as health care use as determined from insurance data, were excluded.

**Figure 1.**
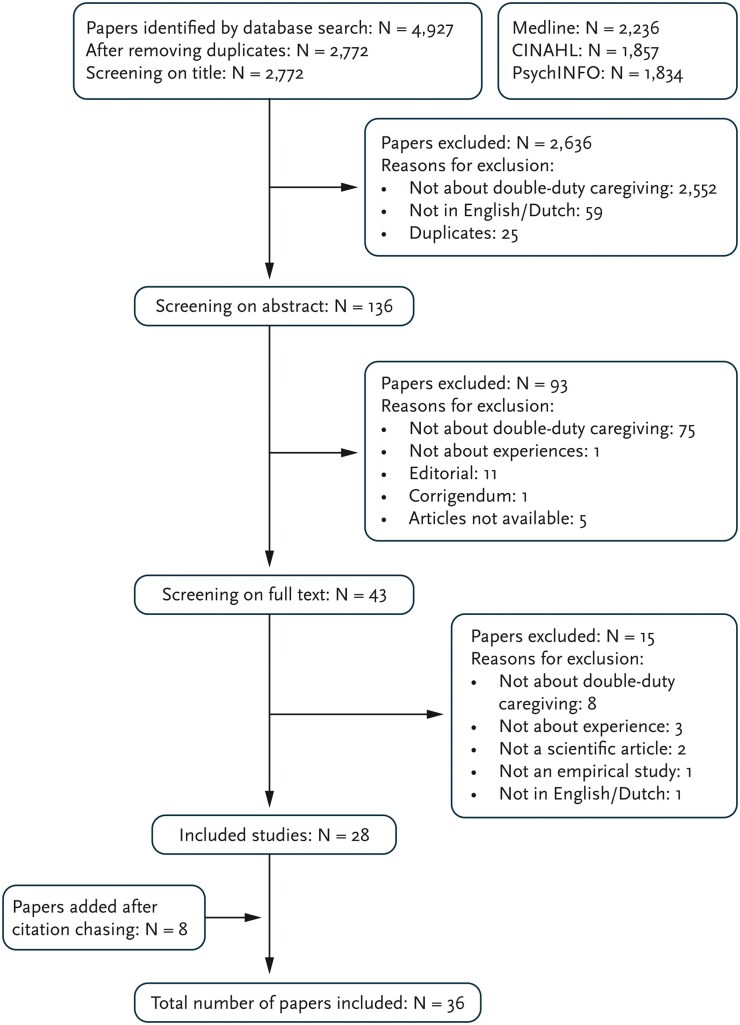
Flow diagram of selection process.

The first author (H.M.) reviewed the articles by screening the titles, and two authors (H.M. and either L.S. or A.S.) screened the abstracts independently for relevance. Full-text screening was also conducted by two authors (H.M. and either L.S. or A.S.). Every disagreement was discussed until an agreement was reached. If no agreement was reached, the articles were discussed among the research team (H.M., L.S., A.S., R.G.). In addition, citation-chasing was carried out. The articles that remained after the screening on title, abstract and full text, were used with both backward citation-chasing (using their reference list) and forward citation-chasing (to see which articles cited that article) to find additional relevant articles. The articles that emerged were screened for their titles, abstracts, and full text, as detailed earlier. Figure 1 shows the process and outcomes.

### Data extraction

To extract the data from the included articles the research team developed and discussed a data-extraction sheet (see online supplementary material Appendix A). Two authors (H.M. and either L.S. or A.S.) completed a data-extraction sheet for each article. By employing an inductive approach, we classified our findings into referenced terms. In total, 80 terms were found, such as “advise other health care workers based on experiences as double-duty caregiver,” “advocate,” “guilt,” “knowledge,” “manager support,” “monitoring,” and “seeking inclusion.” These terms were discussed and grouped by the research team. Our analyses resulted in the following three themes: (1) “Professional background,” (2) “Activities” and (3) “Consequences” as is shown in Table 1.

**Table 1.** Terms and themes.

Themes	Terms				
**Professional background**	Affection	Reasons to work	Knowledge	Expectations	Using connections and positions
	Seeking inclusion	Understands what can go wrong	Burden of knowing	Building relations to get information	Knowledge gives realistic expectations of the care
	Positive relationships to seek inclusion	Not being let in, increasing anxiety	Trust and confidence in the nursing staff	Sharing or not of the professional background	Little opportunity to take unplanned leave
	Unable to take time off	Extra pressure because of labor shortages	Make family sacrifices for the sake of work	Dominant focus in their life	Conflicting expectations and obligations
**Activities**	Watch over their family member	Monitoring/assessing	Coordination of care	Be there (constantly)	Advocate
	Communication with health care workers	Mediate between health care and ill relative	Help in making difficult decisions	Take over the care	Responsible to be assertive
	Advice/guide	Collaboration	Consult/coach/teach		
**Consequences**	Positive effect on family relations	Support from family	Dreams and outlooks were different now	Poor mental health	Poor physical health
	Stress	Fear/anxiety	Burn out	Sleep issues	Guilt
	Higher self-rated burden	Self-doubt	Emotional exhaustion	Lack of energy/fatigue	Lack of time
	Struggle to meet needs of loved one and family	Psychological distress	Loss of social life	Partner relation quality	Hide emotions because of family
	Interference between work and private life	Blurring boundaries	Levels of perceived family time adequacy	Inform manager	Manager support
	Inform colleagues	Help of colleagues	Negativity from colleagues	Presenteeism	Making an error
	Setting limits to informal care	Setting boundaries between work and private life	Positive effects on professional practice	Advise other health care workers	Parttime work
	Flexible hours	Flexibility	Search particular unit, shift or manager	Leaving the job/unpaid leave	Needs for psychological support
	Low schedule control	Less job satisfaction	Difficulties going back to work	Greater psychological job demands	Double-duty caregiving is not seen as a working problem
	Lower ratings of the ability to combine work and informal caregiving	Dissatisfaction on responses to their needs			

## Results

To systematically present the lived experiences of double-duty caregivers, the findings of this study are organized in this section using a narrative style supplemented with tables. First, the results of the literature search and the characteristics of the included studies are described. Subsequently, the results regarding the theme “Professional Background” are presented, consisting of four subthemes. Following this, the theme “Activities” is detailed. Within this theme, we identify and outline seven distinct activities that were consistently referenced across the included articles. The focus then shifts to the impact of being a double-duty caregiver in the theme “Consequences.”

### Study retrieval

Through the selection strategy, a total of 4,927 articles were identified (see Figure 1). After duplicates were removed, 2,772 remained. The screening for abstracts and full texts identified 28 relevant articles. Eight articles were added through citation-chasing. Eventually, 36 articles were included in this study.

### Characteristics of the included studies

Table 2 displays the characteristics of the included studies. Of the 36 articles, 13 studies were conducted in the United States, eight in Canada, six in Australia, four in the Netherlands, one in Sweden, one in Lebanon, one in the United Kingdom, one in Switzerland, and one in Norway.

**Table 2.** Characteristics of included articles.

	Country	Aim of the study	Method	Number and profession of participants
**Qualitative studies**				
**Anjos et al., 2012**	Canada	To explore how gendered expectations and exemptions affect the caregiving experiences and personal health of male nurses caring for family members.	Qualitative secondary analysis of interviews	28, Registered nurses
**Basley et al., 2024**	United States	To explore the experience of double-duty caregivers and to contribute to nursing practice knowledge.	Interviews	10 Registered nurses
**Bristol et al., 2021**	United States	To describe the experiences of health care professionals assuming the role of informal caregiver during intra-hospital transitions of older adults.	Interviews	6, Registered nurses (*N* = 4), certified nursing aid (*N* = 1) and, social worker (*N* = 1)
**Carlsson et al., 2016**	Sweden	To describe how health care professionals understand the role of being a health care professional and a family member of a patient admitted to hospital.	Interviews	18, Registered nurse 9, Physician 2, Assistant nurse 1, Midwife 1, Social welfare officer 2, Occupational therapist 1, Radiographer 1, Biomedical technician 1
**Cicchelli & McLeod, 2012**	Canada	To explore lived experiences of five nurses caring for family members living with advanced cancer.	Interviews	5, Registered nurses
**Detaille et al., 2020**	The Netherlands	To investigate the expectations and needs of double-duty caregivers in the Netherlands, and to examine the meaning of self-management in managing work–life balance.	Focus groups	17, Most were working as nurses
**Giles & Williamson, 2015**	Australia	To understand and interpret the experiences of nurse family members when a family member or loved one is hospitalized in a critical condition.	Interviews	19, Registered nurses
**Heitink et al., 2017**	The Netherlands	To describe the factors that informal carers who are employed in health care organizations identify as affecting their quality of life, labor participation and health.	Interviews	16, Carer, certified nursing assistant, nurse, receptionist, hostess, front desk employee, volunteer coordinator, activities coordinator, operations supervisor and manager
**Jones et al., 2021**	United States	To explore the lived experiences of registered nurses family caregivers during an adult family member’s episode of care in the southern United States.	Interviews	25, Registered nurses
**Khatib et al., 2022**	Lebanon	To describe the experiences of critical care nurse family members when a loved one is admitted to a critical care unit at the Hotel-Dieu de France hospital.	Interviews	6, Registered nurses
**Klages et al., 2020b**	Australia	To examine how health professionals who were mothers had negotiated care for their adult children with schizophrenia within the mental health system.	Interviews	13, Mental health nurses 4, registered general nurses 5, social worker 1, occupational therapist 1, medical researcher 1, medical doctor 1
**Klages et al., 2020a**	Australia	To understand the complex interplay between being a health professional playing critical roles in health care and being a mother of a person requiring mental health care—of being the mother and also having that extra professional knowledge.	Interviews	13, Mental health nurses 4, registered general nurses 5, social worker 1, occupational therapist 1, medical researcher 1, medical doctor 1
**Lines et al., 2015**	Australia	To better understand the specific challenges and needs of nurse–parents whose children were hospitalized for acute illnesses.	Interviews	6, Registered nurses
**Mills & Aubeeluck, 2006**	United Kingdom	To explore the information needs, support systems and impact this experience has upon the nurse’s quality of life to further understand the impact of this dual role.	Interviews	5, Nurses
**Quinney et al., 2018a**	Australia	To understand the personal lifeworld of nurses who care for family members with a chronic illness.	Interviews	15, Nurses
**Quinney et al., 2018b**	Australia	To develop insights into health care provision from understanding the lived experience of participants who were family carer and professional nurse.	Interviews	15, Nurses
**Rosenfeld, 2007**	United States	To describe the caregiving experiences and assess the extent to which informal caregiving is a factor considered by nursing personnel in their decisions to remain or leave hospital employment and whether this varies by job classification, socioeconomic status, cultural, or family arrangements of the nurse; and To develop workplace practices and policy recommendations aimed at retaining family caregivers for the nursing and health care administration at the research site and the wider nursing community.	Interviews	28, Nurse assistant 4, staff nurse 10, advanced practice nurse 8, and leadership 5
**Salmond, 2011**	United States	To explore the experience of being a nurse family member of a relative hospitalized for a critical illness.	Interviews	22, Nurse 12, manager 4, educator 6
**Santerre-Theil et al., 2022**	Canada	To explore the experiences of health professionals who also provide informal support to a family member with cancer.	Interviews	12, Nurses 7, physicians 5
**St-Amant et al., 2014**	Canada	To examine specific negotiating strategies employed by double-duty caregivers in the provision of care to older relative.	Interviews	32, Registered nurses
**Ward-Griffin et al., 2005**	Canada	To examine nurses’ experiences in providing care to older relatives.	Interviews	15, Nurses
**Ward-Griffin et al., 2015**	Canada	To extend our understanding of the social processes of double-duty caregiving and how boundary blurring changes over time (oscillation) within the three double-duty caregiving prototypical experiences (making it work, working to manage, and living on the edge) identified in our previous study.	Interviews	32, Nurses
**Ward-Griffin, 2004**	Canada	To examine the experiences of women in four different health professions (nursing, medicine, physiotherapy, and social work) who provided care to older relatives.	Interviews	37, Nurses 15, physicians 6, physiotherapists 7, social workers 9
**Ward-Griffin et al., 2011**	Canada	To examine compassion fatigue among those nurse–daughter caregivers who were identified as “living on the edge.”	Interviews	20, Nurses
**Wohlgemuth et al., 2015**	United States	To examine the dual roles of such professionals, the impact of their geriatrics expertise on the care of family members, and the influence of those caregiver experiences on their clinical practice.	Interviews	16, Nurses 12, physicians 3, social worker 1
**Quantitative studies**				
**Baumblatt et al., 2022**	United States	To examine the characteristics of nurses who are double-duty nurse family caregivers and the impact the double-duty has on nurse well-being and employment.		257, Nurses 206, managers 51
**Boumans & Dorant, 2014**	The Netherlands	To compare the work-related experiences and personal health status of double-duty caregivers with those of caregivers who do not provide informal care to a family member or close friend in need.	Questionnaire	328, Care function (registered of licensed practical nurse) 90,2%, paramedical 2,5%, nurse specialist 7,3%,
**DePasquale et al., 2018a**	United States	To examine how certified nursing assistants (CNAs) with unpaid family caregiving roles for children (“double-duty-child caregivers”), older adults (“double-duty-older adult caregivers”), and both children and older adults (“triple-duty caregivers”) differed from their nonfamily caregiving counterparts (“workplace-only caregivers”) on four work strain indicators (emotional exhaustion, job satisfaction, turnover intentions, and work climate for family sacrifices). The moderating effects of perceived family time adequacy were also evaluated.	Questionnaire	972, Certified nursing assistants
**DePasquale et al., 2016b**	United States	To examine the psychosocial implications of double-duty child care (child care only), double-duty-older adult care (older adult care only), and triple-duty care (both child care and older adult care or “sandwiched” care).	Questionnaire	397, Health care employees
**DePasquale et al., 2018b**	United States	To examine how women who combine long-term care employment with unpaid, informal caregiving roles for children (double-duty-child caregivers), older adults (double-duty-older adult caregivers), and both children and older adults (triple-duty caregivers) differed from their workplace-only caregiving counterparts on workplace factors related to job retention (i.e., job satisfaction and turnover intentions) and performance (i.e., perceived obligation to work while sick and emotional exhaustion). The moderating effects of perceived spouse support were also examined.	Questionnaire	546, Health care employees, direct care
**DePasquale et al., 2018c**	United States	To investigate the frequency with which women employed in U.S.-based nursing homes entered and exited unpaid caregiving roles for children (double-duty-child caregivers), adults (double-duty-older adult caregivers), or both (triple-duty caregivers), as well as examined how combinations of and changes in these caregiving roles related to cross-sectional and longitudinal sleep patterns.	Questionnaire	135, Health care employees, direct care employees
**DePasquale et al., 2018d**	United States	To examine subjective stress appraisals and perceived schedule control among men employed in the long-term care industry (workplace-only caregivers) who concurrently occupied unpaid family caregiving roles for children (double-duty-child caregivers), older adults (double-duty older adult caregivers), and both children and older adults (triple-duty caregivers).	Questionnaire	125, Health care employees, working in direct care
**Dorant & Boumans, 2016**	The Netherlands	To examine whether employed informal caregivers differ from non-caring colleagues with respect to negative and positive spillover effects, health and work-related outcomes, use of formal support arrangements and experiences with a supportive work environment.	Questionnaire	820, 321 Working in a care company; 499 working in a finance company
**Häusler et al., 2017**	Switzerland	To assess the impact of informal caregiving on burnout risk among health professionals and whether this relationship is mediated by work-privacy conflict or differs between occupational groups.	Questionnaire	1406, Nurses 854 (including midwives), medical doctors 231, other health professionals (including medical-therapeutic experts, medical-technical experts, academic staff) 321
**Scott et al., 2006**	United States	To compare the level of stress and fatigue among full-time hospital staff nurses who provided care for older family members with the levels reported by full-time hospital staff nurses with and without children younger than 18 years living at home. Additional goals included comparing sleep duration and work performance of hospital staff nurses who cared for older family members with those of nurses with and without children living at home, and those caring for both parents and children.	Logbook	393, Nurses
**Stain et al., 2020**	Norway	To explore the frequency of mental health professionals being carers for relatives with mental health problems and in particular the satisfaction of this group of carers with the mental health services.	Questionnaire	453, Bachelor/master health science 230, assistant/nursing aid 67, psychologists 62, doctor/psychiatrist 30, other 53

**Table 3.** Illness, relation, and intensity of care.

Included articles	The illness situation of the care receiver	The social relation between caregiver and receiver	Intensity or duration of care
**Qualitative studies**			
**Anjos et al., 2012**	^a^	Family, most were sons or sons in law	1–21 hr per week, 24% providing 2 hr per week.
**Basley et al., 2024**	Family member at the end of life	Family	from 6 weeks to 1 year
**Bristol et al., 2021**	Relative in an acute care setting, hospital	Older adults/family	^a^
**Carlsson et al., 2016**	Cancer (8), orthopedic/surgery/gynecology (5), Stroke (3) Hospital	Family	Some stayed only a few days, while others had spent a long time in hospital
**Cicchelli & McLeod, 2012**	Living with advanced cancer, pancreatic, lymphoma, colon, lung, and breast cancer.	Family, most ill parents (in law), one aunt	^a^
**Detaille et al., 2020**	^a^	^a^	^a^
**Giles & Williamson, 2015**	Hospitalized with a critical illness	Daughter (in law): 8; sibling (5); mother (2), partner (2); other (2)	^a^
**Heitink et al., 2017**	Cancer (6), autism (4), Mentally retarded (3), and others	Son(s) (9), mother (7), father (6), daughter (3); husband (2); other (2)	^a^
**Jones et al., 2021**	Hospitalized in the past 5 years	Parent (10); spouse (9), grandparent (2), mother/father-in-law (2); others (2)	^a^
**Khatib et al., 2022**	Critical sick	Family	^a^
**Klages et al., 2020b**	Schizophrenia	Adult children	^a^
**Klages et al., 2020a**	Schizophrenia	Adult children	^a^
**Lines et al., 2015**	Acute illness: Pneumonia, pyloric stenosis, burn, appendicitis, thrombosis, bronchiolitis (2), infective arthritis, asthma, vomiting, and dehydration	Children aged 12 or younger	^a^
**Mills & Aubeeluck, 2006**	Diagnosed with a life-threatening illness	Family	^a^
**Quinney et al., 2018a**	Family member with periods of acute exacerbation of a chronic illness: mental health, cardiac disease, autoimmune diseases, and cancers	Family	
Quinney et al., 2018b	Diagnoses including; mental health, hematological disease, autoimmune disease, cardiac disease, and cancer	Family	^a^
**Rosenfeld, 2007**	Dementia, end-stage renal disease, Parkinson’s disease, cancer	Family	^a^
**Salmond, 2011**	Hospitalized for a life-threatening critical illness: heart disease, cancer, and trauma	Adult relative	^a^
**Santerre-Theil et al., 2022**	Cancer	Family	^a^
**St-Amant et al., 2014**	^a^	Older relative	^a^
**Ward-Griffin et al., 2005**	^a^	Parents (66%), parents-in-law (13%), grandparents (7%), sister (7%), spouse (7%)	Weekly care within their own homes from 3 months to 11 years (mean: 5 years)
**Ward-Griffin et al., 2015**	^a^	^a^	^a^
**Ward-Griffin, 2004**	^a^	Most to their parents (in law). Others: grandparents, siblings, friends, aunts and spouses.	^a^
**Ward-Griffin et al., 2011**	^a^	Parents	^a^
**Wohlgemuth et al., 2015**	^a^	Older family member	^a^
**Quantitative studies**			
**Baumblatt et al., 2022**	^a^	Parent 73,3%, partner 21,8%, child 30,1%	^a^
**Boumans & Dorant, 2014**	^a^	Low: parent, child, friend High: parent, child, partner	Made a distinction in low (8 hr and less) and high-intensity (more than 8 hr) care. High intensity: more difficult to combine work and informal care.
**DePasquale et al., 2018a**	Double-duty-older adult caregivers (aging parents with poor or declining health) Double-duty-child caregivers (children under 18, living together 4 days or more) Triple-duty caregivers: both	Parents	^a^
**DePasquale et al., 2016b**	Double-duty-older adult caregivers (adult relative) Double-duty-child caregivers (children under 18, living together 4 days or more) Triple-duty caregivers: both	Adult relative	At least 3 hr a week in the past 6 months
**DePasquale et al., 2018b**	Double-duty-older adult caregivers (adult relative) Double-duty-child caregivers (children under 18, living together 4 days or more) Triple-duty caregivers: both	Adult relative	At least 3 hr a week in the past 6 months
**DePasquale et al., 2018c**	Double-duty-older adult caregivers (adult relative) Double-duty-child caregivers (children under 18, living together 4 days or more) Triple-duty caregivers: both	Older adults	At least 3 hr a week in the past 6 months
**DePasquale et al., 2018d**	Double-duty-older adult caregivers (adult relative) Double-duty-child caregivers (children under 18, living together 4 days or more) Triple-duty caregivers: both	Older adults	At least 3 hr a week in the past 6 months
**Dorant & Boumans, 2016**	^a^	^a^	Low intensity informal caregiving group; less than 8 hr care High intensity informal caregiving group; 8 hr of more informal care
**Häusler et al., 2017**	An adult person in need of care	An adult living outside of the household or living in the same household.	^a^
**Scott et al., 2006**	^a^	Children, older adults, or children and older adults	^a^
**Stain et al., 2020**	51.66% for a relative with mental health problems: depression (34.8%); emotional/behavioral problems (16.3%)	Parent (26.4%); child (23.8%); sibling (16.7%); partner (6.6%)	^a^

a This subject is not addressed in the article.

Of the 36 articles, most were published after 2015. Only 15 articles were published between 2000 and 2015. All 36 selected articles are in English. In several studies, specific types of health care professionals were the target group. In 16 articles, this was (registered) nurses, and in one article, nursing assistants. In four articles (by the same author(s)), the target group is described as “health care employees, working in direct care.” In 17 studies, the target group comprises several disciplines within health care organizations, such as managers, physicians, social workers, and doctors.

To provide a deeper understanding of the caregiver’s situation across the included studies, Table 3 contains information regarding the illness situation of the care receiver, the social relation between caregiver and care receiver, and the intensity or duration of care. With regard to the illness situation, in many articles care receivers with severe and/or life-threatening illnesses are involved and the care receivers are sometimes hospitalized. Care typically involves family members and in some articles a specific family member is mentioned. In most articles, the intensity of care is not specified.

### Professional background

The first theme “Professional Background,” consists of four subthemes: *professional knowledge, expectations, using professional connections and position*, and *information*.

#### Professional knowledge

The first subtheme is *professional knowledge*. Professional knowledge is an important theme and is mentioned in half the articles. Double-duty caregivers work as health care professionals and therefore possess relatively much professional knowledge of health care, which distinguishes them from other informal caregivers (Anjos et al., 2012; Giles & Williamson, 2015; Salmond, 2011). Their knowledge includes professional knowledge about the diagnosis of their ill relatives, the standards of the needed care and treatment, and how the health care system works (Cicchelli & McLeod, 2012; Heitink et al., 2017; Jones et al., 2021; Mills & Aubeeluck, 2006; Quinney et al., 2018a, 2018b).

Professional knowledge gives double-duty caregivers a sense of confidence and relief (Bristol et al., 2021; Lines et al., 2015), and helps them obtain the best treatment and care for their ill relatives (Carlsson et al., 2016; Khatib et al., 2022; Klages et al., 2020a, 2020b; Lines et al., 2015; Mills & Aubeeluck, 2006; Quinney et al., 2018b; Rosenfeld, 2007; St-Amant et al., 2014; Wohlgemuth et al., 2015). They feel well equipped to guide their ill relatives (Santerre-Theil et al., 2022). Their ­professional knowledge also brings them fear of knowing the dangers associated with the condition and anxiety about the unknown (Quinney et al., 2018a, 2018b).

#### Expectations

The second subtheme in the theme “Professional Background” is *expectations*. Due to their strong feelings of affection for their ill relatives (Ward-Griffin, 2004; Ward-Griffin et al., 2011; Wohlgemuth et al., 2015) combined with professional knowledge and skills, health care professionals expect themselves to be in charge when a relative gets ill (Basley et al., 2024; Bristol et al., 2021; Detaille et al., 2020; Giles & Williamson, 2015; Jones et al., 2021; Khatib et al., 2022; Ward-Griffin, 2004; Ward-Griffin et al., 2005; Wohlgemuth et al., 2015). Some double-duty caregivers feel that they are the only ones who can provide informal care the right way because of their professional background (Detaille et al., 2020; Heitink et al., 2017) or feel that they have little or no choice about becoming an informal caregiver for their relatives (Ward-Griffin, 2004; Ward-Griffin et al., 2005; Wohlgemuth et al., 2015). Moreover, the ill relatives become the focus of their lives (Lines et al., 2015; Quinney et al., 2018a, 2018b; Salmond, 2011), and everything else becomes a secondary concern (Salmond, 2011). They believe that harm might come to their relative if they are not involved in their care (Ward-Griffin et al., 2005; Ward-Griffin et al., 2011).

As the “nurse in the family,” double-duty caregivers’ families also expect them to be in charge when a care situation occurs (Basley et al., 2024; Detaille et al., 2020; Giles & Williamson, 2015; Jones et al., 2021; Ward-Griffin et al., 2005), to inform and advise the ill relative (Quinney et al., 2018a) as well as their family (Anjos et al., 2012; Cicchelli & McLeod, 2012; Khatib et al., 2022; Quinney et al., 2018a; Santerre-Theil et al., 2022). The involvement of double-duty caregivers has a calming effect on the family (Salmond, 2011). Additionally, their involvement is attributed to their gender, as women are traditionally expected to take on the role of caregiver for an ill relative (Ward-Griffin et al., 2005). Wohlgemuth et al. (2015) mention the influence of the special position of some double-duty caregivers in their families because they are, for instance, the only child, work part-time, and/or live nearby (Wohlgemuth et al., 2015). They can struggle to meet the needs of both their ill relatives and their other family members (Klages et al., 2020a) and hide their emotions because of family needs (Khatib et al., 2022; Salmond, 2011). On the other hand, the care situation can lead to a closer relationship between the double-duty caregivers and their ill relative (Quinney et al., 2018a).

In addition to the expectations of themselves and their family members, double-duty caregivers must deal with the expectations of the health care organization from which their relative receives care (Basley et al., 2024; Detaille et al., 2020). They often feel the moral duty to take over caregiving tasks because of the nurses’ workload in care for older adults, as they are very aware of the shortage of health care professionals (Detaille et al., 2020; Ward-Griffin et al., 2005) and the heavy workload within health care organizations (Lines et al., 2015). Staff expects double-duty caregivers to carry out nursing tasks (Giles & Williamson, 2015; Jones et al., 2021; Quinney et al., 2018a; Ward-Griffin, 2004; Ward-Griffin et al., 2011) even when they lack the necessary professional knowledge (Quinney et al., 2018a).

#### Using professional connections and position

The third subtheme in the theme “Professional Blackground” is *using professional connections and position*. Because of their work as health care professionals, double-duty caregivers use their professional connections and position to get advice about the situation of their ill relative and achieve the best care and treatment for their relative (Anjos et al., 2012; Jones et al., 2021; Quinney et al., 2018a, 2018b; Stain et al., 2020; Ward-Griffin et al., 2005), especially when the expectations of care for the ill relative are not being met (Cicchelli & McLeod, 2012). Having these connections and position makes them ­different again from other informal caregivers (Jones et al., 2021). They have the skills to navigate the health care system (Bristol et al., 2021; Carlsson et al., 2016; Jones et al., 2021; Quinney et al., 2018b; Santerre-Theil et al., 2022) and learn about appropriate standards of care (Quinney et al., 2018b).

#### Information

The fourth and final subtheme in the theme “Professional Background” is *information*. Information has a direct link with the professional knowledge mentioned earlier. Double-duty caregivers have (more or less) professional knowledge but not all the information they might want and need about the actual situation of their relatives. They want specialized and tailored information to monitor the situation of their relative and advocate for them (Bristol et al., 2021; Carlsson et al., 2016; Giles & Williamson, 2015; Khatib et al., 2022; Lines et al., 2015; Stain et al., 2020).

Double-duty caregivers become their family’s *de facto* ­educators (Khatib et al., 2022). To get this information, they seek inclusion in the health care organization. They work the system, build positive relationships with staff to gain access (Bristol et al., 2021; Giles & Williamson, 2015; Quinney et al., 2018b; Salmond, 2011), and must be assertive to get the necessary information (Bristol et al., 2021). They also use their professional colleagues as sources of information (Salmond, 2011; Ward-Griffin et al., 2005; Ward-Griffin et al., 2011).

In addition, family members expect double-duty caregivers to have and share information, primarily about the illness and treatments, but also about the illness process (Cicchelli & McLeod, 2012; Giles & Williamson, 2015; Quinney et al., 2018b).

### Activities

Double-duty caregivers offer a wide range of activities within the caregiving context for their relatives. In this study, within the theme “Activities,” the seven activities are outlined that are referenced in the included articles.

#### Monitoring

*Monitoring* or *assessing* is an activity for double-duty caregivers. They use their professional knowledge and experience to monitor their relatives, e.g., to oversee, evaluate, and examine the (medical) condition of their relatives (Basley et al., 2024; Bristol et al., 2021; Carlsson et al., 2016; Klages et al., 2020b; Lines et al., 2015; Quinney et al., 2018a; Santerre-Theil et al., 2022; Ward-Griffin et al., 2015) and evaluate the care provided (Bristol et al., 2021). They also monitor the mental condition of the ill relative (Klages et al., 2020b).

#### Watching over

*Watching over* their ill relatives is also an activity of double-duty caregivers (Giles & Williamson, 2015; Quinney et al., 2018b), as they are constantly watchful and alert (Quinney et al., 2018a, 2018b; Santerre-Theil et al., 2022) and want to be there for their ill relatives (Lines et al., 2015). This activity is seen as second nature to double-duty caregivers (Salmond, 2011).

#### Advising

Besides advising family members, double-duty caregivers also provide their ill relatives with information and instruction on questions the ill relative should ask the doctors and claims to make (Carlsson et al., 2016). They advise, coach, and guide their ill relatives in dealing with the health care system (Santerre-Theil et al., 2022; St-Amant et al., 2014). Furthermore, double-duty caregivers advise their ill relatives in making decisions: for instance, about the needed care or treatment (Basley et al., 2024; Cicchelli & McLeod, 2012; Santerre-Theil et al., 2022; Ward-Griffin et al., 2005).

#### Advocating

Double-duty caregivers *advocate* for their ill relatives (Basley et al., 2024; Carlsson et al., 2016; Giles & Williamson, 2015; Klages et al., 2020a, 2020b; Santerre-Theil et al., 2022; Ward-Griffin, 2004; Ward-Griffin et al., 2015), especially when they notice errors or deficiencies or when improvements in treatment or care are possible (Bristol et al., 2021; Lines et al., 2015; Salmond, 2011; Ward-Griffin et al., 2005). They advocate especially when ill relatives are not in the condition to make decisions themselves (Santerre-Theil et al., 2022; Ward-Griffin et al., 2005, 2011). Double-duty caregivers in a leadership position use this position to get things done to improve the care for their ill relative (Rosenfeld, 2007). They can feel disempowered when dissatisfied with the care delivered to their relative or because of the responses to their own needs (Mills & Aubeeluck, 2006).

#### Collaborating

*Collaboration* by double-duty caregivers encompasses both working together as a family on behalf of their ill relative (St-Amant et al., 2014) and cooperating and communicating with the professional caregivers involved (Basley et al., 2024; Klages et al., 2020b). They are seen as the mediator between health care professionals and the ill relative (Quinney et al., 2018b).

#### Coordinating care

Carlsson et al. (2016) describe several double-duty caregivers who felt that they had to coordinate fragmented care. In addition, Ward-Griffin et al. (2005) state that double-duty caregivers coordinate care: for instance, by educating their family members and supervising the care they provide. *Coordinating care* means focusing on the outcome and is based on the double-duty caregivers’ professional knowledge (St-Amant et al., 2014). Several articles describe coordinating care as a strategy to address the care situation (St-Amant et al., 2014; Ward-Griffin, 2004), and this coordination is expected from double-duty caregivers, as they are “the nurse in the family” (Ward-Griffin et al., 2015). Part of the coordination, as well as a coping strategy, is the delegation of necessary care from them to the other family members (Anjos et al., 2012; Rosenfeld, 2007; St-Amant et al., 2014; Ward-Griffin, 2004; Ward-Griffin et al., 2005).

#### Caregiving

Depending on the situation of the ill relative, double-duty caregivers provide more or less *direct care*, such as physical and socioemotional care (Basley et al., 2024; Klages et al., 2020b). The characteristics of the double-duty caregivers also influence the extent to which informal care is provided. An example is presented by Anjos et al. (2012) who found that male double-duty caregivers delegate care more often and teach other members of the family about caregiving rather than providing care considered not “gender appropriate” themselves. Moreover, double-duty caregivers feel that they must take over the caregiving if the care provided is insufficient or unsafe (Carlsson et al., 2016) to make sure that the ill relative receives the best care possible (Ward-Griffin, 2004).

### Consequences

Following the themes “Professional Background” and “Activities,” the “Consequences” of being a double-duty caregiver is the third and final theme of this study. In this theme, the following subthemes have been delineated: *blurring boundaries*, *interference and conflict*, *personal consequences*, *consequences at work*, and *coping strategies*.

#### Blurring boundaries

One consequence of caregiving as a professional and in one’s personal life is *blurring boundaries*. Double-duty caregivers move between their family role and their nursing role (Cicchelli & McLeod, 2012; Jones et al., 2021; Wohlgemuth et al., 2015). On one hand, they are health care professionals with professional knowledge seeking information, monitoring, advising, and coordinating, as described. On the other hand, they are, for instance, also mothers, partners, or children of an ill relative. They experience both clinical responsibilities and human concern for their ill relative (Khatib et al., 2022) and manage and balance multiple roles (Basley et al., 2024). They also feel pressure from their family and the professionals of the health care organization involved to enact their nurse identity (Santerre-Theil et al., 2022), and they have difficulties switching off their nurse role (Giles & Williamson, 2015). Especially when the ill relative is treated in the hospital where the double-duty caregivers work, it is more difficult for them to cope with their double role (Santerre-Theil et al., 2022). Sometimes, the roles cease to be separate and become intertwined (Klages et al., 2020b; Lines et al., 2015; Mills & Aubeeluck, 2006) or conflicting (Cicchelli & McLeod, 2012; Quinney et al., 2018a); this can lead to self-doubt (Ward-Griffin et al., 2015).

As a result, the boundary between the two roles can blur (Jones et al., 2021; St-Amant et al., 2014; Ward-Griffin et al., 2015). The more double-duty caregivers are involved in the situation of a ill relative, the more often the boundaries blur (Ward-Griffin, 2004; Ward-Griffin et al., 2005). Trusting and gaining confidence in the health care organization involved with one’s ill relative is needed to allow their family role to emerge (Salmond, 2011). Blurring boundaries can lead to negative health experiences (St-Amant et al., 2014), like additional burden (Cicchelli & McLeod, 2012), stress (Ward-Griffin et al., 2015), and physical and mental fatigue (Ward-Griffin et al., 2005). Because of these intertwining roles and the expectations double-duty caregivers experience when disclosing their nursing role, some want to hide their nursing selves (Giles & ­Williamson, 2015; Jones et al., 2021; Khatib et al., 2022). Disclosure is seen as necessary for their concerns to be taken seriously by the health care organization that cares for the ill relative (Lines et al., 2015).

#### Interference and conflict

Besides blurring boundaries, another consequence is related to the interference of double-duty caregivers’ work life and private life. Boumans and Dorant (2014) studied the differences among health care professionals involved in non-, low-, and high-intensity informal caregiving. The results indicate that when (more) time is spent on informal care tasks, double-duty caregivers feel less able to combine these tasks with their work. DePasquale et al. (2018d) separated respondents into health care professionals who had no care tasks, cared for a child, cared for parents, or cared for both (triple-duty caregivers) and found that triple-duty caregivers experienced more work–family conflict than other groups. Häusler et al. (2017) studied the conflict between work life and private life among the same four groups and found that the score for work–home conflict was a predictor for burnout scores in both double-duty caregivers and triple-duty caregivers.

#### Personal consequences

Providing informal care has implications for the personal lives of double-duty caregivers. Therefore, *personal consequences* is the next subtheme within the theme “Consequences.” Double-duty caregivers report positive outcomes of being both health care professionals and informal caregivers, as is mentioned in the context of the theme “knowledge.”

Besides these positive experiences, many double-duty caregivers suffer from stress (Anjos et al., 2012; Basley et al., 2024; Baumblatt et al., 2022; Cicchelli & McLeod, 2012; DePasquale et al., 2016; Klages et al., 2020a; Lines et al., 2015; Scott et al., 2006; Ward-Griffin et al., 2015; Wohlgemuth et al., 2015) or emotional exhaustion (Basley et al., 2024; Boumans & ­Dorant, 2014; DePasquale et al., 2018a, 2018b, 2018d; ­Heitink et al., 2017; Khatib et al., 2022; Klages et al., 2020a; Lines et al., 2015; Salmond, 2011; Ward-Griffin, 2004; Ward-Griffin et al., 2015; Wohlgemuth et al., 2015).

Double-duty caregivers experience professional knowledge as a source of stress (Cicchelli & McLeod, 2012), and stress occurs when the health care organization shares “privileged” information with them but not with other family members (Cicchelli & McLeod, 2012). Double-duty caregivers also experience fear and anxiety (Giles & Williamson, 2015; Heitink et al., 2017; Lines et al., 2015; Salmond, 2011) that result from their *professional knowledge*, as described before. Furthermore, the fear that their relative is not receiving the appropriate care or treatment (Klages et al., 2020a)—for instance, when double-duty caregivers must rely on other professionals to care of their relative (Heitink et al., 2017)—causes them to perceive the care situation as an emotional rollercoaster (Khatib et al., 2022). They are overwhelmed and they have expressed the need for emotional support from their network (Basley et al., 2024) and psychological assistance to help them cope with the situation (Klages et al., 2020a). They do not always have the knowledge and skills to stand up for their needs (Detaille et al., 2020).

Being a double-duty caregiver has consequences for one’s mental and physical health (Boumans & Dorant, 2014; Klages et al., 2020a). Besides emotional exhaustion, they also experience physical exhaustion (Ward-Griffin et al., 2015, 2011), psychological distress (DePasquale et al., 2016; Klages et al., 2020a), a lack of energy (Heitink et al., 2017; Ward-Griffin et al., 2011), and a lack of self-care (Basley et al., 2024). Triple-duty caregivers experience mental and physical fatigue (Scott et al., 2006) and have a higher risk of burnout than health care professionals without informal care responsibilities (Häusler et al., 2017). Double-duty caregivers may experience burnout from their caregiving responsibilities (Baumblatt et al., 2022) due to the expectations of others (Anjos et al., 2012) and the perception by their managers that their challenges are not considered work-related problems (Detaille et al., 2020). Double-duty caregivers have a higher self-rated burden compared to health care professionals without their informal care tasks (Dorant & Boumans, 2016). Stain et al. (2020) found that about one in five double-duty caregivers has received treatment for their own mental-health problems.

Guilt about not knowing more (Jones et al., 2021; Ward-Griffin, 2004) and struggling with multitasking (Wohlgemuth et al., 2015) is mentioned as an emotion experienced by double-duty caregivers. It is also an issue when they know that the quality standards of the treatment of care are not achieved or when they are not present at an important moment (Quinney et al., 2018a). In addition, guilt is felt when the health of the relative deteriorates under the care of the double-duty caregivers (Ward-Griffin, 2004).

Double-duty caregivers experience sleeping problems (Baumblatt et al., 2022; Heitink et al., 2017; Ward-Griffin et al., 2011). They have higher rates of sleepiness at work (Scott et al., 2006), sleep less than health care professionals without informal care responsibilities, and have sleep of lower quality and sufficiency (DePasquale et al., 2018c). Lack of time is mentioned as a problem of double-duty caregivers because of their double-care duty. They are constantly either working or caregiving (Basley et al., 2024; Santerre-Theil et al., 2022). As a consequence, their network and social world shrinks, risking their social isolation (Heitink et al., 2017). Triple-duty caregivers experience less support from their partners (DePasquale et al., 2016). They also experience a loss of social life (Baumblatt et al., 2022). For them, life has changed, and their dreams and outlook are now different (Quinney et al., 2018a).

#### Consequences at work

In addition to the *personal consequences*, double-duty caregivers also experience *consequences at work*. For instance, double-duty caregivers who provide more than 8 hr of informal care a week experience presenteeism, caused by, for instance, exhaustion (­Boumans & Dorant, 2014; Dorant & ­Boumans, 2016).

Furthermore, the likelihood of making an error in their professional work is doubled in double-duty caregivers (Scott et al., 2006). Both double-duty caregivers and triple-duty caregivers report facing higher psychological job demands than health care professionals with no informal care responsibilities (DePasquale et al., 2016). They feel more obligated to make family sacrifices for the sake of work than health care professionals without informal care roles (DePasquale et al., 2018a). Triple-duty caregivers also report less job satisfaction (DePasquale et al., 2018a). Double-duty caregivers report that their informal caregiving responsibilities affect their employment status by, for instance, producing a fear of losing their jobs or promotion opportunities (Baumblatt et al., 2022). After a temporary unpaid leave because of their informal care duties, double-duty caregivers find it hard to start working again (Baumblatt et al., 2022). Furthermore, double-duty caregivers are very aware of the shortage of health care professionals at work and, therefore, feel unable to take time off or are uncomfortable doing so (Detaille et al., 2020; Mills & Aubeeluck, 2006; Rosenfeld, 2007; Ward-Griffin et al., 2005).

DePasquale et al. (2018d) studied the influence of schedule control on double-duty caregivers. She concluded that double-duty caregivers with low control over their own schedules experience greater perceived stress, higher turnover intentions, and less job satisfaction compared to double-duty caregivers with high control over their schedules.

Despite its negative aspects, being a double-duty caregiver also has some positive effects on work. Double-duty caregivers draw on their experiences as a family member of an ill relative and provide guidance to other health care professionals in their interactions with informal caregivers of patients (Jones et al., 2021; Khatib et al., 2022). Being a double-duty caregiver themselves, gives them insights into informal caregiving and what it means to be an informal caregiver (Santerre-Theil et al., 2022; Wohlgemuth et al., 2015). After this experience, double-duty caregivers help family members of patients to take care of themselves (Wohlgemuth et al., 2015).

Within the subtheme *consequences at work*, the relationship with the manager and colleagues requires specific attention. According to Boumans and Dorant (2014) and Dorant and Boumans (2016), most double-duty caregivers inform their managers and colleagues about their informal care roles. Still, some do not inform their manager, because they do not feel the need to discuss the their informal caregiving tasks (Dorant & Boumans, 2016). Even though one’s manager is not always informed, the attitude of the manager is important for double-duty caregivers. An understanding manager helps them to cope with the situation of being both a formal and informal caregiver (Ward-Griffin et al., 2005; Ward-Griffin et al., 2011). From the double-duty caregivers point of view, managers tend not to see the combination of professional and informal caregiving as a topic for conversation with their employees (Detaille et al., 2020; Heitink et al., 2017), as sustainable employability is not addressed in the organization (Heitink et al., 2017). Double-duty caregivers mention that the support from their managers is limited, referring to the possible arrangements of the organization, like unpaid leave (Baumblatt et al., 2022).

Besides one’s manager, co-workers are also important to double-duty caregivers. They can support them (Basley et al., 2024; Baumblatt et al., 2022; Heitink et al., 2017; Salmond, 2011; Ward-Griffin et al., 2005, 2011), cover their shifts, and rearrange their schedules (Ward-Griffin et al., 2005) and may be a source of information about the informal care situation. In addition, double-duty caregivers experience negativity from co-workers. Baumblatt (2022) presents the example of a double-duty caregiver who points out that empathy is often lacking in relation to long-term issues and that the act of taking leave has resulted in adverse reactions within the team.

#### Coping strategies

An additional aspect of the theme consequences pertains to the *coping strategies* employed by double-duty caregivers. To manage their complex lives, double-duty caregivers have various coping strategies, categorized in *flexibility*, *using workplace arrangements* and *setting limits*. *Flexibility* at work is seen as an important coping strategy (Basley et al., 2024): the ability to leave work for emergencies (Rosenfeld, 2007; Ward-Griffin et al., 2005), take unpaid leaves of absence (Ward-Griffin et al., 2005, 2011), change the work shift (Basley et al., 2024), and delegate their responsibilities at work to others (Rosenfeld, 2007). Another coping strategy is to seek out particular work departments, shifts (e.g., only day or night shifts), or supervisors who enable the combination of their responsibilities in providing both formal and informal care (Heitink et al., 2017; Rosenfeld, 2007). Part-time employment is also identified as a coping strategy to have more time available for ill relatives (Ward-Griffin, 2004).

Another coping strategy can consist of *utilizing workplace arrangements* or services for employees. However, such arrangements or services at work are reported as either unknown, not perceived as available, inconvenient, ineffective, or inaccessible to double-duty caregivers (Rosenfeld, 2007). Dorant and Boumans (2016) found that double-duty caregivers who provide more than eight hours of care a week to their relative make more use of arrangements than other employees: For instance, flexible hours and part-time work (Dorant & Boumans, 2016). Double-duty caregivers tend to solve problems directly with their colleagues instead of utilizing arrangements (Heitink et al., 2017). Also, establishing personal limits can be seen as a form of coping with the informal care situation (Ward-Griffin, 2004; Ward-Griffin et al., 2005, 2015), for instance, by coordinating, delegating, and supervising the informal care (Ward-Griffin et al., 2005) and limiting family expectations (Ward-Griffin et al., 2015). Another way for double-duty caregivers to cope with the informal care situation is creating a strict boundary between one’s public and private spheres, with no disturbance from their private life allowed during working hours (Rosenfeld, 2007).

## Discussion

The aim of this scoping review was to investigate what is known in the scientific literature about the experiences of double-duty caregivers. In the studied literature, we found three themes: “Professional background,” “Activities,” and “Consequences.” Within the three themes, positive aspects of being a double-duty caregiver are identified, such as the underlying affection, the confidence gained through knowledge, the positive impact on family relations, and the support provided by colleagues. Within the theme “Professional background” the subtheme *professional knowledge* seems to be a central issue. It is likely that because of their professional knowledge, double-duty caregivers themselves and others expect double-duty caregivers to provide informal care for their ill relatives. Some activities appear to be based on this professional knowledge, for instance, they monitor the ill relative’s health, advise and advocate for their ill relative, and coordinate and provide their care. The positive and negative consequences double-duty caregivers experience are likely to be a result of their professional knowledge. A beneficial outcome seems to be that their knowledge gives them confidence. Conversely, negative outcomes may include the blurring of boundaries between their two roles, as well as fear of knowing the dangers.

Nowadays the responsibility to care for an ill relative is expected to shift toward the social network (Broese van ­Groenou & De Boer, 2016; de Klerk et al., 2021; Sacco et al., 2022; Vos et al., 2021; ZonMw, 2024). This will have significant implications for all informal caregivers.

Examining their tasks, several of double-duty caregivers’ responsibilities align with those of informal caregivers in general, and some are different. Informal caregivers in general provide, most of all, practical help, such as household care, transportation to doctors, social visits, and support with arranging formal care (Broese van Groenou & De Boer, 2016; Ngo et al., 2025; Vos et al., 2021). In addition, medical tasks, personal care, coaching, and the coordination of care are provided by informal caregivers (Plaisier et al., 2015; Vos et al., 2021). It is likely that double-duty caregivers, due to their knowledge, more often monitor, advice and advocate their ill relatives.

For all informal caregivers, support at work is critical (Santerre-Theil et al., 2022; Stain et al., 2020). A high level of support seems to improve the balance between work and informal caregiving (Plaisier et al., 2015), prevents informal caregivers from developing depressive feelings (Bijnsdorp et al., 2022), and seems to improve work outcomes (Plaisier et al., 2015). According to caregivers, informal caregiving should be recognized as a shared responsibility between employer and employee (Vos et al., 2021). This recognition, support, and acknowledgement are also suggested for double-duty caregivers (Anjos et al., 2012; Baumblatt et al., 2022; Detaille et al., 2020; Giles & Williamson, 2015; Heitink et al., 2017).

The suggestions in the literature about coping with double-duty caregivers’ double-duty focus mainly on the organizations where double-duty caregivers work. These organizations should be supportive (Ward-Griffin et al., 2005), for instance, by giving them a reliable and emotionally safe environment, listening to them (Khatib et al., 2022), and addressing their needs (Anjos et al., 2012). In addition, support services at work, like training, advice, and counseling, can help double-duty caregivers (Baumblatt et al., 2022; Rosenfeld, 2007). As managers are important for them, we also suggest that managers should be trained to address the double-duty care situation (Rosenfeld, 2007) and support double-duty caregivers to take time for themselves (Detaille et al., 2020). It should be of great importance to employers to ensure a good work–life balance for their employees, as the consequences of this balance are significant, both for work and private life (Tomaževič et al., 2014).

In addition to the possible coping strategies coming from the organizations where double-duty caregivers work, coping strategies can also be provided by three other sources: by the double-duty caregivers themselves, by the network of the ill relatives and the double-duty caregiver, and by the health care organization providing care to the ill relatives. Double-duty caregivers should acknowledge the risk of becoming overburdened and take proactive steps to prevent it. One example would be arranging (additional) home care, assistance, or support—either for themselves or for their loved one. However, in light of current labor shortages in the health care sector, arranging additional formal care or support is likely to be challenging. Being a team with others from the social network of the ill relative and the double-duty caregiver is important to relieve them. Nevertheless, it is essential to acknowledge also the limits of this approach to avoid overload of the other involved caregiver(s). Knowledge on the collaboration and mutual expectations and understanding between the double-duty caregiver, the other members of the social network of the ill relative and the double-duty caregiver and the organization that provides formal care to the ill relative is still ­limited. Further research is needed to deepen the understanding of this collaboration.

## Strengths and limitations

This scoping review provides new insights into the experiences of double-duty caregivers. To the best of our knowledge, this research has not been previously done. Due to our structured methodology, using search terms and subsequent snowballing, we did the best we could to minimize the chance of missing relevant references.

The study does have some limitations. First, the studies are from several countries (details are provided in Table 2) and cultures and may differ in terms of health care facilities and facilities for employees. These differences may have influenced the results. The second limitation concerns the article selection process. As the screening based on titles was performed by only one researcher, there is a risk of selection bias. Thirdly, our study was conducted from the perspective of the experiences of double-duty caregivers. Consequently, alternative perspectives—such as that of social determinants–were not examined. Conducting the study from a different perspective might have led to different findings. Fourthly, there are several differences in focus among the included studies. The studies define different target groups. In some studies, the only participants were (registered) nurses or nursing assistants. In other studies, different disciplines were involved besides nurses: for instance managers. Some studies did not specify the target group, referring to the participants only as “health care employees in direct care.” The results in these studies are not described per target group. Thus, from these studies no conclusions can be drawn on characteristics and outcomes per type of health care professional. A third difference involves the ill relative. Some studies specifically concern providing informal care for older adults, a relative with cancer, or a critical illness, or a child with schizophrenia. Some studies specifically describe the experiences of double-duty caregivers with a relative who is hospitalized. Finally, one difference concerns the nature of the illness. Some articles specifically describe the experience of double-duty caregivers who provide informal care for a relative with an acute disease or a relative with a long-term illness, disability, or condition. In our study, we searched for experiences of double-duty caregivers concerning their double role. In doing so, we did not differentiate by target group or the specific circumstances of the ill relative. These differences may well affect the double-duty caregivers’ experiences, as, for instance, the experience of providing informal care to a relative with an acute, life-threatening illness may differ from that of providing long-term care to a relative with a disability or chronic condition.

## Implications for practice and research

Given the aging global population and the transition from formal to informal care, double-duty caregivers will face increasing expectations and therefore the road to exhaustion, burnout, sick leave, or even resignation may be short. They should be aware of these increasing expectations, set limits, and arrange support for themselves. Given the current labor market shortages, it is crucial for employers to support double-duty caregivers in maintaining a good quality of life to ensure their continued participation as a health care professional. The organization that provides formal care to the ill relative should also be aware of the double-duty care situation of double-duty caregivers.

Our study shows that much is known about the knowledge of double-duty caregivers, the expectations placed on them, their use of their connections, and the personal consequences of their double-care duty. However, several gaps remain. More research is needed about how double-duty caregivers experience their work and the impact of the double-care duty on their work, for instance, disclosing caregiving responsibilities to one’s manager and acknowledging the potential impact of these responsibilities on work performance, including the risk of errors. Also, the available and needed support system for double-duty caregivers at work and in their private lives, and their strategies for successfully managing their double-care duty are largely unknown. Subsequently, insights can be developed about policy and tools that can help double-duty caregivers maintain balance between their double-care duties.

## Conclusion

This study shows that experiences of double-duty caregivers are diverse and influenced by multiple factors, including their professional background. Because of their professional knowledge, self-imposed and external expectations arise, double-duty caregivers perform various roles in caring for their ill relatives, such as monitoring, advocating, and advising. In their personal lives, double-duty caregivers experience consequences of their double-care duty: for instance, stress and exhaustion. They also mention positive aspects of their double-care duty, such as learning from their experiences, educating their colleagues, and helping informal caregivers at work. To cope with their double-care duty, double-duty caregivers mention that they need a flexible, understanding, and supportive work environment.

Because of the higher demand placed both now and in the future on informal caregivers in general and on double-duty caregivers specifically, double-duty caregivers should learn to set limits on both their own expectations, the expectations of others and arrange (additional) support if available. Additional support from the employer is necessary to secure their engagement as employees. Finally, support from a formal caregiver, who provide care for their ill relatives, is crucial. Further research is needed to map the experiences of double-duty caregivers with and the consequences of their double-care duty at work, the available and needed support for them (both privately and at work), and strategies for successfully managing their double-care duty. Finally, research should be conducted about policy and tools that can help double-duty caregivers stay balanced.

## Supplementary Material

gnaf238_Supplementary_Data

## Data Availability

No original data were generated for this study. However, the literature that was included in the review and the analytic methods are available to other researchers and can be accessed by contacting the corresponding author. The study has been preregistered, and the registration can be accessed via the following link: https://doi.org/10.17605/OSF.IO/7NZT3.
